# A New Case of DRESS Syndrome Induced by Sulfasalazine and Triggered by Amoxicillin

**DOI:** 10.1155/2013/409152

**Published:** 2013-07-10

**Authors:** Francesco Girelli, Simone Bernardi, Lucia Gardelli, Bruna Bassi, Gianluca Parente, Alessandra Dubini, Luigi Serra, Maurizio Nizzoli

**Affiliations:** ^1^Internal Medicine Department, Rheumatology Unit, G.B. Morgagni Hospital, Via Forlanini 34, 47121 Forlì, Italy; ^2^Dermatology Unit, G.B. Morgagni Hospital, Via Forlanini 34, 47121 Forlì, Italy; ^3^Pathology Unit, G.B. Morgagni Hospital, Via Forlanini 34, 47121 Forlì, Italy

## Abstract

Drug Rash Eosinophilia Systemic Symptoms (DRESS) syndrome is a systemic hypersensitivity reaction characterized by exfoliative dermatitis and maculopapular rash, lymphadenopathy, fever, eosinophilia, leukocytosis, and involvement of internal organs as liver, lung, heart, and kidney; the disorder starts within 2–6 weeks after taking a drug with an incidence that ranges from 1/1000 to 1/10000 exposures. Fatal cases are reported. The exact pathogenesis of DRESS syndrome is not completely understood, while it is reported that amoxicillin could trigger it in patients who are taking allopurinol, sulfasalazine, NSAIDs, carbamazepine, strontium ranelate, lisinopril, lansoprazole, and minocycline. Amoxicillin could act directly, inducing the reactivation of a viral infection (HHV 6 and EBV) with symptoms similar to DRESS syndrome or by reducing the patients' ability to detoxify the body from substances chronically taken. We describe a case of a patient admitted to our hospital for a DRESS syndrome flared after amoxicilline intake during treatment with sulfasalazine; this combination can activate severe reactions often with an insidious onset that can mimic an infectious disease.

## 1. Case Report

A 53-year-old woman, Caucasian, without history of drug intolerance, heavy smoker, affected by remote diagnosis of Lyme disease, previous removal of ovarian cyst, and gastroesophageal reflux, was diagnosed in another hospital as having a seronegative spondyloarthritis with anterior right uveitis. For this reason sulfasalazine was started until the dosage of 2 grams per day; after six weeks of treatment, amoxicillin/clavulanic acid was administered for the onset of sore throat, fever and laterocervical lymphadenopathy, and sulfasalazine suspended. After 3 days, she was admitted to our hospital for an acute diffuse and itchy rash with hemorrhagic vesicles on oral cavity, facial edema, and worsening of lymphadenopathy and fever. On admission mental status was normal, body temperature was 39.2°C, blood pressure was 130/80, and the heart and respiratory rates were, respectively, 115 beats and 22 acts per minute.

Laboratory tests showed neutrophilic leukocytosis with mild eosinophilia (WBC: 12200/mmc, PMNn: 8580/mmc, and Eo: 690/mmc), AST: 106 IU/L (nv < 30 IU/L), ALT: 350 IU/L (nv < 30 IU/L), GGT: 1047 IU/L (nv 5–36 IU/L), alcaline phosphatase: 2959 IU/L (nv < 240 IU/L), total bilirubin: 2,71 mg/dL (nv < 1.0 mg/dL), C-reactive protein 112 mg/L, (nv < 5.0 mg/L), and ERS 103 mm/1 hour; serum creatinine, glucose, calcium, Na eK, TSH, total gamma globulins, IgG/A/M, K/Lambda chain ratio, and INR were normal.

Amoxicillin/clavulanic acid was suspended, and the patient switched to ceftriaxone and levofloxacin association for suspected sepsis.

Blood and urine cultures resulted normal; serologic tests for an acute infection by EBV, parvovirus B19, CMV, HCV, HBV, HIV, spirochete, *Borrelia burgdorferi*, *Salmonella* and *Shigella*, *Chlamydia trachomatis*, *adenovirus*, *Enterovirus*, *Leptospira*, *Bartonella*, herpes simplex, Simplex V2, and Zoster, resulted negative according to the cutoffs of our laboratory. Among autoantibodies, rheumatoid factor, antinuclear antibodies, antiENA, antiDNA, ANCA, anticardiolipin, and lupus anticoagulants were negative.

A CT scan with and without contrast showed right pleural effusion, diffuse thoracic and abdominal lymph nodes enlargement (maximum diameter 26 mm), severe hepatomegaly, mild splenomegaly, and no signs of pneumonia. A transesophageal echocardiogram excluded cardiac valve vegetations and pericardial effusion. A bone biopsy from the iliac crest excluded lymphoma or hematologic malignancy, revealing mild eosinophilic hyperplasia. Skin and liver biopsies showed nonspecific results, consisting, respectively, in chronic perivascular infiltration, made mostly by lymphocytes with very rare eosinophils, and a portal tracts infiltration of lymphohistiocytosis, neutrophils and eosinophils with intralobular foci of necrosis.

On the third day of hospitalization, the clinical status quickly worsened for severe deterioration of skin rash and of heart and respiratory rates; once we received the results of coltures from blood sample, antibiotics were suspended and methylprednisone 1 mg/Kg/day was started, with prompt recovery of fever, rash, lymph nodes enlargement, and laboratory abnormalities. The diagnosis of DRESS syndrome was made, and the patient was discharged with the indication to gradually reduce corticosteroid dosage. About four months after discharge the patient had recurrence of arthralgias and right uveitis, without clinical signs of arthritis and/or enthesitis; according to the ESSG classification criteria for seronegative spondyloarthritis [1], the previous diagnosis was not confirmed, and cyclosporine-A, 3 mg/Kg/day, was administered for the recurrence of the ocular disorder. One year later, the clinical outcome was favourable; cyclosporine-A was definitely suspended, after gradual reduction of the daily doses. After three years, the patient was followed for symptoms related to osteoarthritis and fibromyalgia.

## 2. Discussion

DRESS syndrome is a drug hypersensitivity reaction, potentially fatal in up to 10% of cases, whose incidence ranges from of 1/1000 to 1/10000 drug exposures [2]. The hallmark features of DRESS syndrome, not always detectable at the same time, include diffuse maculopapular rash, facial edema, lymphadenopathy, fever, eosinophilia, lymphocytosis, and internal organs involvement [3]. Even if no gold standard is available, some authors have proposed diagnostic criteria based upon clinical and laboratory findings [4, 5]; tachycardia, leucocytosis, and tachypnea during early stage disease, coagulopathy and gastrointestinal bleeding during maximal stage disease have been identified as independent prognostic variables [6]. DRESS syndrome was firstly described in patients treated with anticonvulsants (hence the acronym AHS: Anticonvulsant Hypersensitivity Syndrome); later, it became clear that many other drugs, some of which commonly used by rheumatologists (sulfasalazine, allopurinol, and NSAIDs) [7], could be responsible of the symptoms of DRESS; on the basis of these findings some authors prefer the acronym DIDM-OHS (Drug Induced Delayed Multi-organ Hypersensitivity Syndrome) than DRESS, in which eosinophilia is inconstantly detected [8]. 

The pathogenesis of  DRESS syndrome is not well clarified yet; amoxicillin and other common antibiotics have been observed to correlate with flares of the disorder, in patients taking sulfasalazine [9–11]. In the reported cases, patients were taking sulfasalazine for 4–8 weeks, and the introduction of amoxicillin for a suspected upper respiratory tract infection was followed after 3-4 days by the symptoms of DRESS; something similar has been described during chronic treatment with anticonvulsants [12]. Other commonly used *β*-lactams have been reported to be related, alone or in combination with other drugs, to DRESS syndrome that flared from 16 to 28 days after the starting of the treatment [13, 14]. How amoxicillin can trigger DRESS syndrome is cause of debate: it has been demonstrated that the drug can induce reactivation of HHV 6 and EBV [15, 16] even in patients taking sulfasalazine [17]. In their description of a fulminant hepatic failure, Mennicke et al. outlined that the patient, who was taking sulfasalazine for about 6 weeks, presented the flare of DRESS syndrome few days after having switched from amoxicillin to vancomycin for a resistant *Staphylococcus aureus* systemic infection; also in this case the authors found laboratory signs of an acute HHV 6 infection/reactivation [18]. Also naproxen, ibuprofen, and allopurinol have been associated with an *in vivo* HHV 6 reactivation-enhanced replication [7]. On the contrary, in a case of DRESS syndrome induced by bosentan (an endothelin receptor antagonist with a sulphonamide group, approved for pulmonary arterial hypertension and to prevent the recurrence of new digital ulcers in patients affected by systemic sclerosis), serologic test for acute HHV 6 infection was negative [19].

In the cases related to amoxicillin, two observations are notably: the fact that the symptoms of DRESS syndrome flared not immediately after the introduction of the antibiotic, as it happens in anaphylactic reactions [20] but 2-3 days later, and the possibility that the reason for which the antibiotic was prescribed was not an upper respiratory tract infection but just the early clinical manifestation of DRESS. An external agent, amoxicillin itself or viral reactivation, might affect, through an immune-mediated mechanism or a direct toxic effect, a response against the components of the cytochrome P450 enzymes, reducing their ability to detoxify substances already present in the organism [21]. It has been suggested, in patient taking lamotrigine, that the body's ability to metabolize the drug might be overwhelmed when its threshold concentration is too high [22]. Something similar could have happened in our patient who, after taking sulfasalazine for about 40 days, presented the symptoms of DRESS following an apparently nonspecific febrile infection of the upper respiratory tract, treated with amoxicillin/clavulanic acid, a fact which has been already described by other authors [23]. As we have not taken the tests for infection HHV 6, we cannot be sure of what has been the precipitating event, an acute infection, or the antibiotic given to treat it, or a combination of them that could affect the ability of the organism to metabolize sulfasalazine.

The useful clinical information that our case would suggest is that many drugs, often used by rheumatologists, may result in severe systemic reactions, potentially fatal, sometime resembling sepsis [24]; the systemic framework is often preceded by nonspecific manifestations as fever, sore throat, lymphadenopathy, and rashes, which are interpreted as nonspecific infections of the upper respiratory tract and subsequently treated with antibiotics. We recommend caution in interpreting seemingly minor symptoms, always keeping in mind the drug history of patients taking chronic therapies and the temporal sequence of events.
